# Genome‐wide DNA methylome analysis reveals methylation subtypes with different clinical outcomes for acute myeloid leukemia patients

**DOI:** 10.1002/cam4.3291

**Published:** 2020-07-06

**Authors:** Haiyan Gao, Xin He, Qiang Li, Ying Wang, Yaoyao Tian, Xi Chen, Jinghua Wang, Yan Guo, Wei Wang, Xiaoyun Li

**Affiliations:** ^1^ Department of Hematology The Second Affiliated Hospital Harbin Medical University Harbin China; ^2^ Department of Paediatrics The Second Affiliated Hospital Harbin Medical University Harbin China; ^3^ Assessment Admittance Section Heilongjiang Hospital Service Management Evaluation Center Harbin China

**Keywords:** acute myeloid leukemia, clinical prognosis, methylation, subtype

## Abstract

Leukemia is the second common blood cancer after lymphoma, and its incidence rate has an increasing trend in recent years. Acute myeloid leukemia (AML) is one of the prevalent forms of leukemia. Although previous studies have investigated the methylation profile for AML patients, the AML methylation subtypes based on the genome‐wide methylome are still unclear. In the present study, we identified three methylation subtypes for AML samples based on the methylation profiles at CGI, CGI shore, CGI shelf, and opensea genomic contexts. Analyzing the molecular characteristics and clinical factors of the three subtypes revealed different methylation patterns and clinical outcomes between them. Further analysis revealed subtype dependent marker genes and their promoter CpG sites with regulatory function. Finally, we found that combining the AML patient age and methylation pattern brought better clinical outcome classification. In conclusion, we identified AML methylation subtypes and their marker genes, these results may help to excavate potential targets for clinical therapy and the development of precision medicine for AML patients.

## Graphical abstract

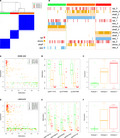

## 1 INTRODUCTION

Acute myeloid leukemia (AML) is an aggressive hematopoietic stem cell malignant clone disease and the most common type of acute leukemia in adults, caused by the accumulation of immature leukemic blasts in the blood and bone marrow.^1^ In recent years, significant progress has been made in decoding the molecular genetics and epigenetic basis of AML and in identifying new candidate prognostic biomarkers. In addition, a classification of the AML has been a progressive shift from a morphologic classification scheme to one informed by causative genomic alterations.^2^, ^3^ Further investigation found that an increasing number of genetic changes have been recognized in the new World Health Organization (WHO) classification of AML.^4^ Apart from genetic alterations, epigenetic changes also reflect biological processes associated with disease progression and treatment response.

DNA methylation, one of the most important epigenetic regulatory mechanisms, was an epigenetic process of transformation of cytosine into 5‐methyl cytosine.^5^, ^6^ DNA methylation regulated gene expression by altering chromatin structure, DNA conformation, and DNA stability.^7^, ^8^ CpG islands (CGI) are defined as sequence ranges where the Obs/Exp value is greater than 0.6 and the GC content is greater than 50%. CGIs are typically located in the promoter region, 5′ to the TSS. CpG shores are contexts with lower CpG density that lie within the 2 kb up‐ and downstream of a CpG island. CpG shelves are defined as the 2 kb outside of a shore. CpG openseas are with low methylation and not characterized in any of the above.^9^, ^10^ The phenomenon of DNA chemical modification may occur in CGI, CGI shore, CGI shelf, and opensea genomic contexts.^11^, ^12^ Early studies have found an abundance of epigenetic alterations in various types of AML.^13^, ^14^ However, most studies limited their research in CGI while ignored the genome‐wide methylome. The understanding of methylation pattern variation for AML samples remains incomprehensive. Therefore, we expected to decode the methylation pattern of AML patients on genome‐scale, including CGI, CGI shore, CGI shelf, and opensea genomic contexts.

To decode the genome‐wide methylation pattern for AML patients, we focused on the most variable CpG sites in CGI, CGI shore, CGI shelf, and opensea genomic contexts, respectively, and identified three subtypes based on corresponding four methylation profiles using cluster‐of‐cluster alignment (COCA) method.^15^ This approach took into account genome‐wide methylation patterns and identified the subtypes from a more general perspective compared with only CGIs. Survival analysis for the subtype samples derived from genome‐wide methylation profiles showed more significant difference than that from single regional methylation profiles. Subsequent analyses for AML subtypes further confirmed the molecular and clinical difference between them. These result indicated that integrating genome‐wide methylome to identify methylation subtypes was essential.

## 2 MATERIALS AND METHODS

### 2.1 Data collection

The level 3 CpG methylation in CGI, CGI shore, CGI shelf, and opensea genomic contexts of 140 AML patients detected by Illumina Infinium HumanMethylation450 BeadChip array were obtained from The Cancer Genome Atlas (TCGA) portal (http://cancergenome.nih.gov/). The level 3 mRNA expression profiles, level 4 mutational datasets, and level 3 Copy Number Variation (CNV) data were also derived from TCGA database. The expression of mRNAs was measured as fragments per kilobase of exon per million reads mapped (FPKM). In addition, we obtained clinical characteristics of the patients from TCGA, including survival time, survival state, age, and other information.

### 2.2 The methylation profiles in CGI, CGI shore, CGI shelf, and opensea genomic contexts of AML samples

We extracted the CpG sites in CGI, CGI shore, CGI shelf, and opensea genomic contexts and further constructed four methylation profiles for AML samples. CpG sites with missing values in more than 30% samples were removed and each of the remaining missing value was imputed by the KNN Imputation.

### 2.3 The somatic mutations of AML samples

Somatic mutations of samples sequenced by whole‐exome sequencing were downloaded from the TCGA database. We integrated all sequencing platforms and obtained the no‐redundancy results. After removing silent mutations, we counted the number of somatic mutations to evaluate Tumor Mutational Burden (TMB) for each sample.

### 2.4 Identifying the methylation‐associated subtypes of AML samples

We classified the AML samples using COCA method^15^ based on the genome‐wide CpG methylation profiles. First, the CpG sites in each type of genomic contexts with top 10% variable methylation levels were kept to identify the regional clusters. Second, consensus clustering for the methylation profile in CGI, CGI shore, CGI shelf, and opensea was developed separately for each. Third, clusters defined from each methylation profile were coded into a series of indicator variables for each cluster to construct a Boolean matrix, and then taken as the input of second‐level consensus clustering. Finally, we obtained the subtypes of AML samples based on the genome‐wide methylation profiles. The consensus clustering was performed by ConsensusClusterPlus R‐package.^16^ ConsensusClusterPlus was run with 80% sample and 80% CpG site resampling and 1000 iterations of hierarchical clustering based on a Pearson correlation distance metric.

### 2.5 Survival analysis

Survival analyses were performed to evaluate the difference in survival rate between more than two sample groups, such as AML subtypes and different AML clusters resulting from separate methylation profile in different genomic contexts. The Kaplan‐Meier survival plots, log‐rank tests, and multivariate Cox regression models were performed using the R package ‘survival’.

### 2.6 Functional enrichment analysis

Functional enrichment analysis at the GO and KEGG levels was performed using DAVID Bioinformatics Resources (https://david.ncifcrf.gov/).^17^ The DAVID enrichment analysis was limited to GO‐FAT biological process (BP) terms and KEGG pathways with the whole human genome as background.

## 3 RESULTS

### 3.1 Identification of the AML subtypes based on the genome‐wide methylation profiles

**FIGURE 1:**
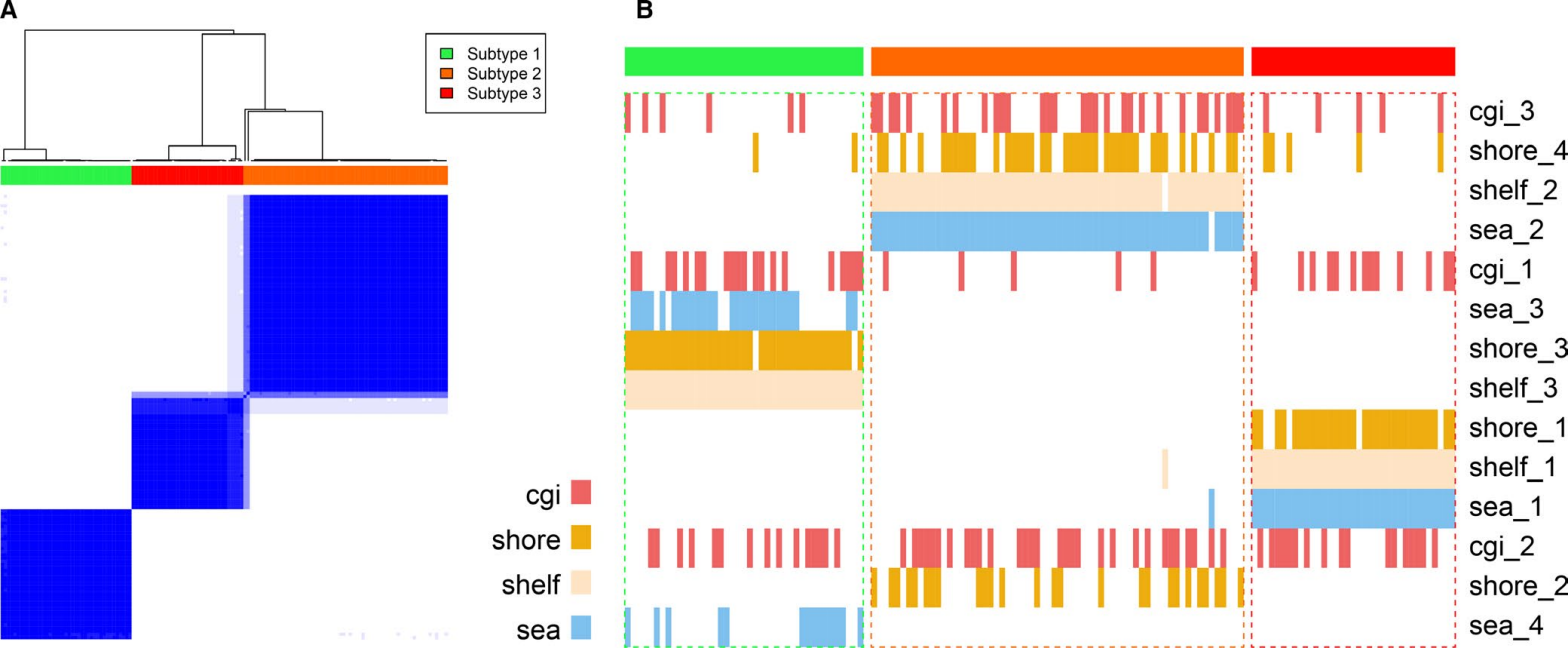
Consensus clustering for AML samples. A, Second‐level consensus clustering for AML samples when K = 3. B, The connection between clusters from consensus clustering of first‐level and second‐level. The matrix of first‐level clusters was further clustered. Each location type of CpG sites is represented by a different color as shown in colorbar

Identification of cancer subtypes make us more understandable about the heterogeneity across cancer samples from the epigenetic perspective and provide potential individualized therapeutic basis.^18^ Therefore, we attempted to identify the AML subtypes with the genome‐wide methylation profiles. To characterize DNA methylation patterns across different genomic contexts in AML samples, we exacted the beta values of CpG sites at CGI, CGI shore, CGI shelf, and opensea contexts separately, and constructed four corresponding methylation profiles. After data pre‐processing described in the ‘Materials and Methods’ section, we extracted the top 10% variable CpG sites, that is, 13 986, 9223, 3145 and 13 226 sites in CGI, CGI shore, CGI shelf and opensea contexts respectively. Next, we identified the AML subtypes based on the four preprocessed methylation profiles, using the method named COCA.^15^ First, 3‐4 clusters were obtained by consensus clustering with each methylation profile. Second, the clustering results from single level were integrated and finally three subtypes were identified as the AML methylation subtypes, with 41, 64 and 35 samples in subtypes 1, 2 and 3 respectively (Figure 1A,B).

### 3.2 Samples in subtype 1 were with better prognosis compared with subtypes 2 and 3

**FIGURE 2:**
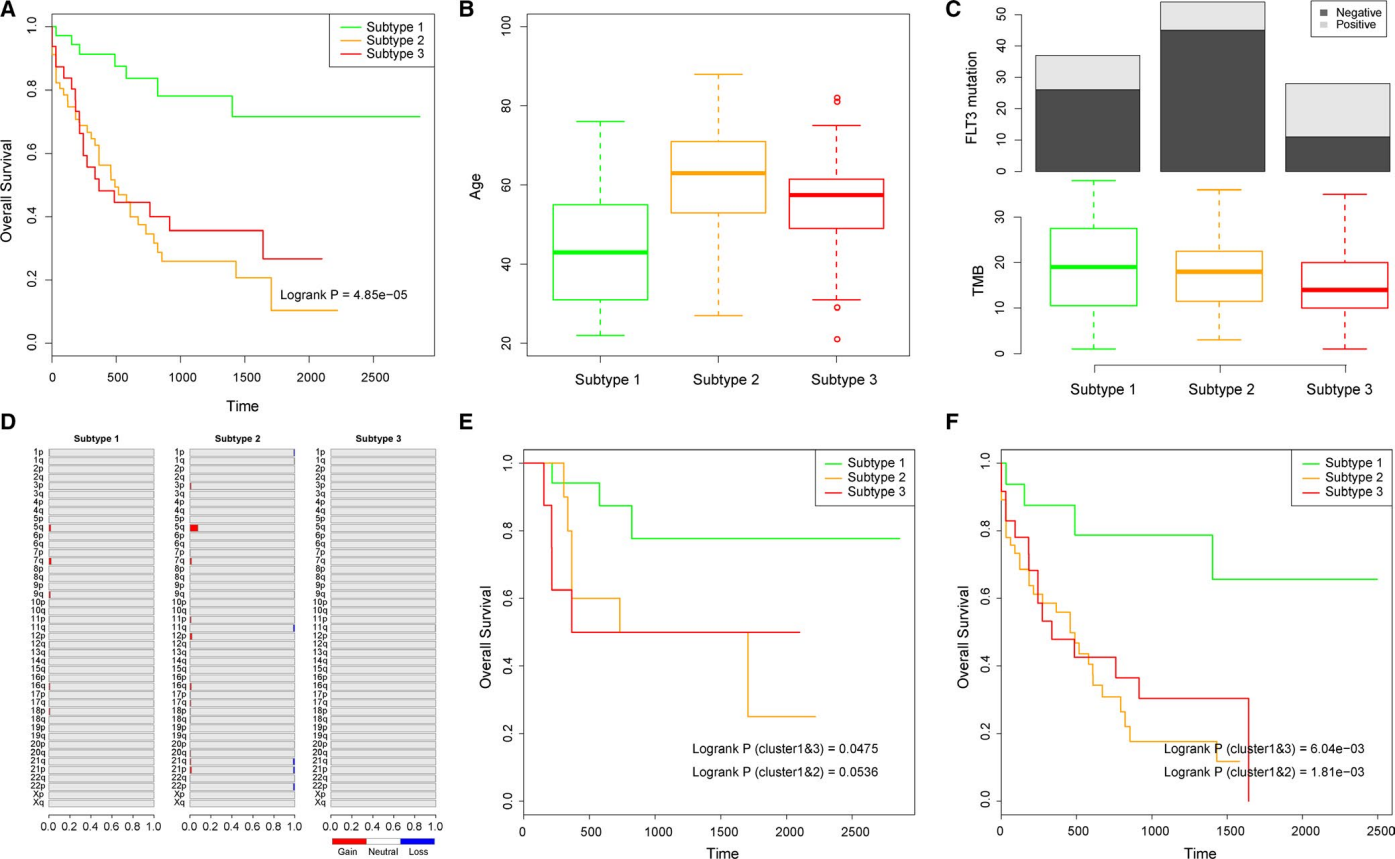
The clinical and molecular difference between patients of three subtypes. A, Kaplan‐Meier survival curves for three methylation subtypes of AML samples. B, Ages of AML patients in three subtypes. C, FLT3 mutation activity and Tumor Mutational Burden (TMB) of AML patients in three subtypes. D, Frequency of arm level alterations in three subtypes. Bar graphs show the frequency of arm level amplifications (red) and deletions (blue). E‐F, Kaplan‐Meier survival curves for younger (E) and elder (F) AML patients

Many studies have shown that variances at the molecular level lead to the different survival rate and different clinical behaviors across samples. It is precisely because of the clinical differences that it is of significance for us to identify the cancer subtypes. Therefore, we compared the survival rate between different AML subtypes. As a result, the samples in subtype 1 showed significantly better prognosis, compared with the other two subtypes (*P* = 4.85e‐05, *P*_(1&2)_ = 6.65e‐06, *P*_(1&3)_ = 2.91e‐04, Figure 2A). Moreover, we found the different clinical behaviors between these three subtypes. Patients in subtype 2 were the eldest, followed by the patients in subtype 3, while those patients in subtype 1 were the youngest (*P*_(1&2)_ = 8.171e‐07, *P*_(1&3)_ = 5.318e‐03, *P*_(2&3)_ = 2.314e‐02, Figure 2B). The result showed that elder patients had worse prognosis than younger patients, this was reasonable and consistent with the results of previous studies.^19^, ^20^, ^21^ Expected for the survival rate and clinical behaviors, we also compared the somatic mutations between the three subtypes. As a result, we found that patients in subtype 3 were with more frequent FLT3 mutations compared with subtypes 1 and 2 (*P* = 2.207e‐02, 1.075e‐04 respectively, Figure 2C). What's more, TMB of subtype 3 samples tended to be less than samples in subtypes 1 and 2 (*P* = .08, .06 respectively, Figure 2C). Then we evaluated the frequency of arm level amplifications and deletions in three subtypes (Figure 2D). The result showed that there was little change in the overall copy number of the leukemia samples, but the samples in subtype 2 and subtype 1 have more amplified and deleted regions compared with subtypes 3. As somatic mutations and CNV represent the genome instability of samples,^22^ we supposed that different methylation levels of samples led to the variant genome instability and finally caused different malignancy grade of AML. Taken together, patients of subtype 2 were eldest and had higher genome instability while patients of subtype 3 were with more FLT3 mutations. These factors were supposed to be the reasons of their poor prognosis. These results suggested that the progression of AML was a complicated procedure that influenced by multiple factors, such as mutation of cancer‐related genes and age of patients.

To investigate whether the prognostic ability of the different methylation patterns was independent of other clinical variables, we performed the multivariate Cox regression analysis. The variables in the regression included subtypes derived from clustering of genome‐wide methylation pattern, age, gender and FLT3 mutation activity. We found that methylation subtypes and age were independent prognostic factors (Table 1). Next, data stratification analysis was performed for age. Patients with a younger (age < 50) and elder age (age ≥ 50) were stratified into younger group and elder group respectively. For younger or elder patients, we further compared the survival rate between three subtypes. As a result, we found that methylation subtype could classify patients within each age stratum into three subtypes with significantly different survival rate (*P*_(1&3, younger)_ = 4.75e‐02, *P*_(1&2, younger)_ = 5.36e‐02, Figure 2E and *P*_(1&3, elder)_ = 6.04e‐03, *P*_(1&2, elder)_ = 1.81e‐03, Figure 2F). This result suggested that the prognostic ability of methylation subtypes was also age‐independent.

**TABLE 1:** Multivariate Cox regression analysis of the subtype classification of AML samples

Variables	coef	HR	95% CI of HR	*P* value
Methylation Subtype				
Subtype 1	Reference			
Subtype 2	1.34	3.83	(1.59‐9.21)	2.71e‐03
Subtype 3	1.32	3.76	(1.50‐9.44)	4.78e‐03
Age	0.05	1.05	(1.03‐1.07)	2.16e‐05
Gender				
Female	Reference			
Male	−0.20	0.82	(0.48‐1.40)	0.469
FLT3 mutation				
Negetive	Reference			
Positive	0.36	1.43	(0.78‐2.63)	0.246

### 3.3 Integrating genome‐wide methylation facilitated the identification of AML subtypes

**FIGURE 3:**
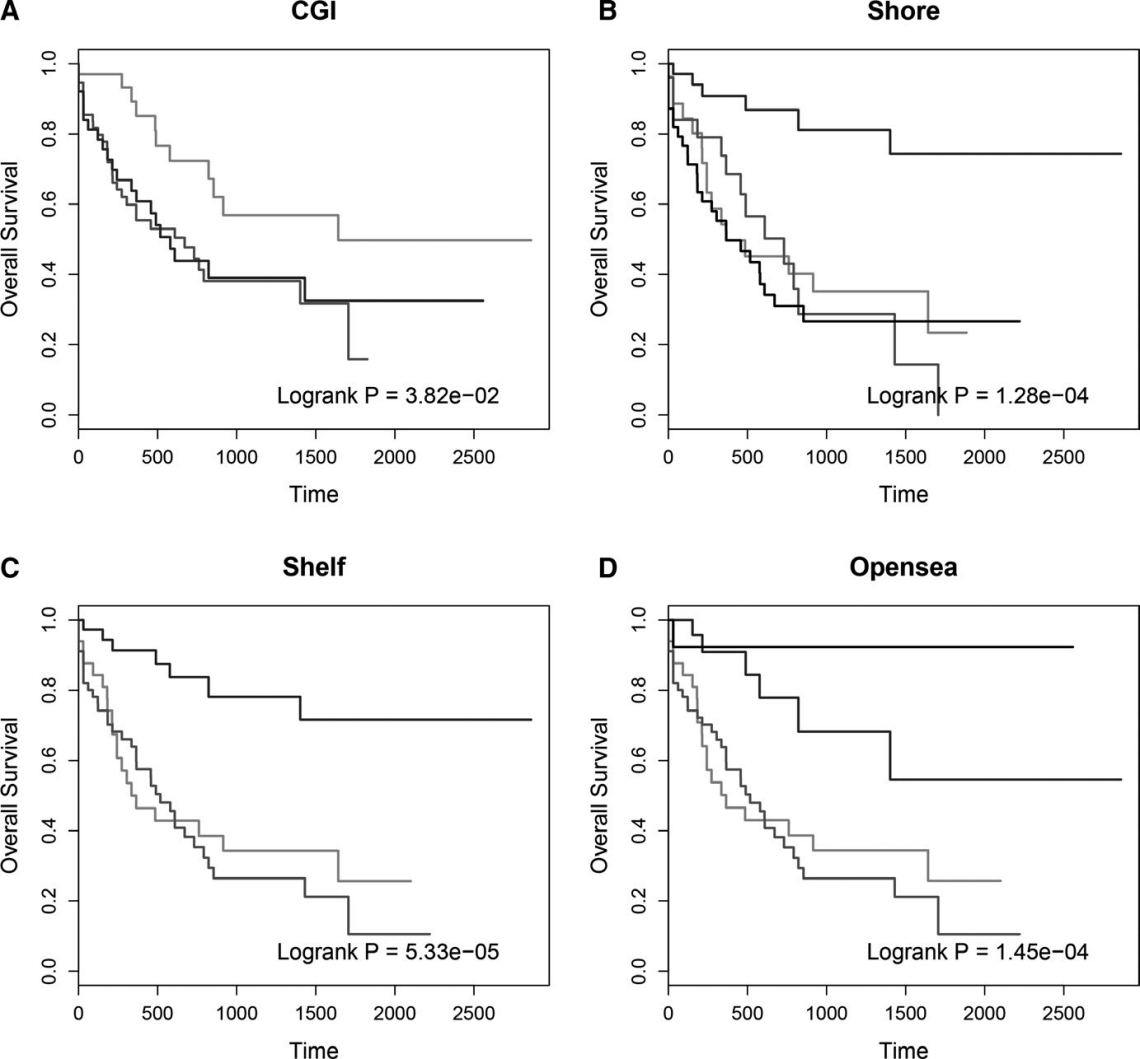
The Kaplan‐Meier survival curves for AML samples of different clusters derived from different locations, including CGI (A), CGI shore (B), CGI shelf (C) and opensea (D)

For the analysis of methylation profiles, most studies focused on the CGIs while ignored the methylation pattern on other genome regions.^23^, ^24^, ^25^ In this study, we used the COCA method to identify the AML subtypes based on the methylation profiles at different genomic contexts, including CGI, CGI shore, CGI shelf, and opensea. As described above, there was significant different survival rate between the three subtypes. Next, we investigated the survival analysis for the three clusters derived from consensus clustering of CGI methylation profile. The result showed that although survival rate were different between these three clusters (*P* = 3.82e‐02), the difference was much less than the three subtypes derived from genome‐wide methylation profiles (Figure 3A). In addition, log‐rank tests for other clustering results derived from methylation profiles at CGI shore, CGI shelf, and opensea were all less significant than subtypes obtained based on genome‐wide DNA methylome (*P* = 1.28e‐04 for shore, 5.33e‐05 for shelf and 1.45e‐04 for opensea, Figure 3B,D). This result suggested that integrating genome‐wide methylation profiles to identify the AML subtypes is necessary and effective. On the other hand, statistic for the samples in different subtypes showed that most samples in each subtype were clustered into same first‐level clusters. This result suggested that samples in the same subtype usually exhibited similar methylation pattern across genome‐wide CpG sites.

### 3.4 Different methylation patterns between three subtypes

**FIGURE 4:**
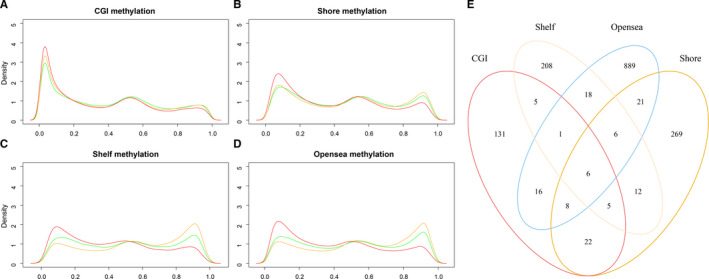
The methylation of CpG sites and differential methylation CpG related genes. A‐D, The density plots for methylation levels of AML subtypes, including CGI (A), CGI shore (B), CGI shelf (C) and opensea contexts (D). E, The overlap across protein‐coding genes that related to significantly differential methylation CpG sites at different genome locations

**FIGURE 5:**
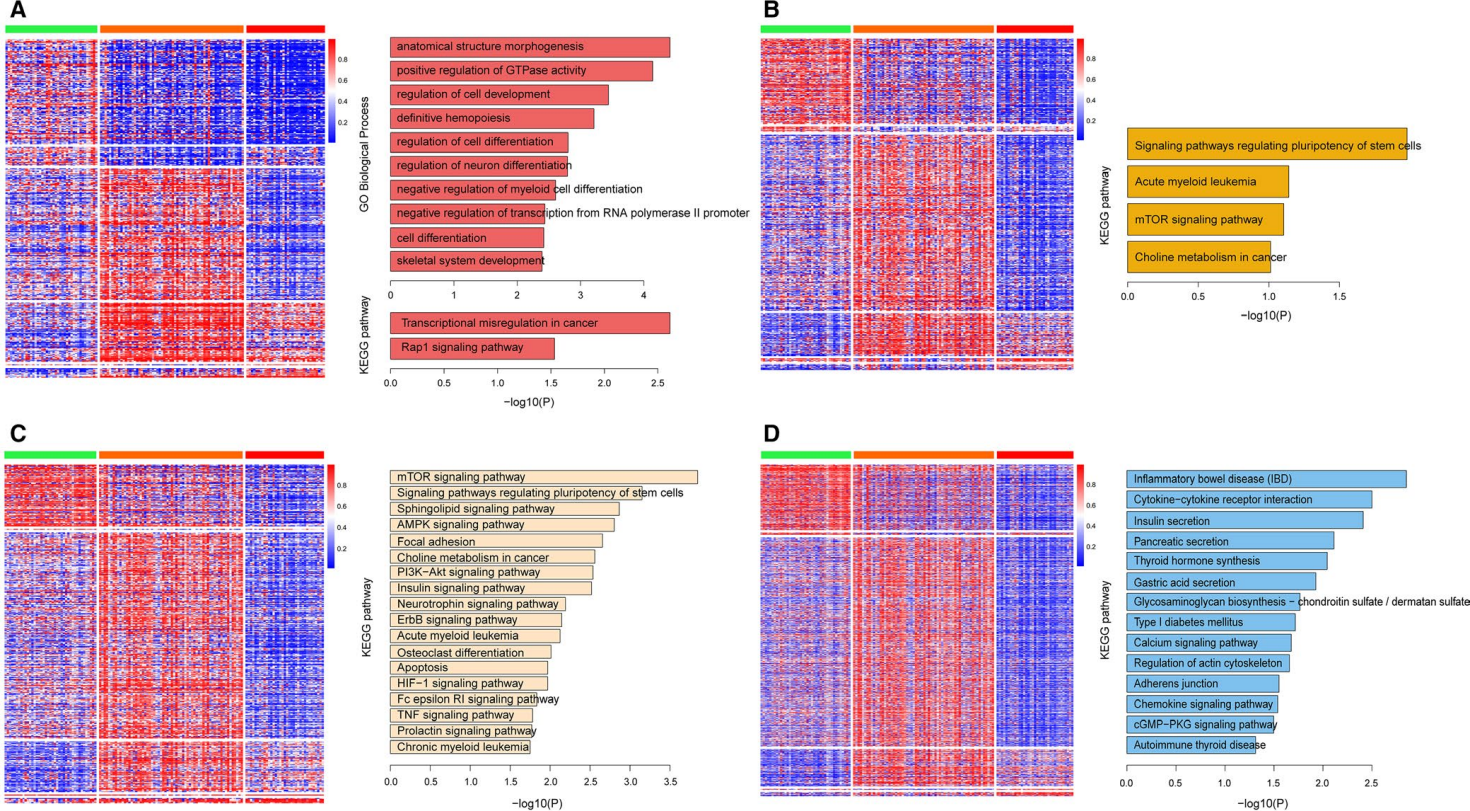
The methylation values of different methylated CpG sites between each of three subtypes and functional enrichment of DMRGs at four types of genomic contexts, including CGI (A), CGI shore (B), CGI shelf (C),and opensea (D)

Next, we analyzed the methylation levels between three subtypes at different genomic contexts. The result showed that samples of subtype 3 were genome‐wide hypomethylation compared with those samples of subtypes 1 and 2 (Figure 4A‐D). We also found that samples of subtype 2 showed the highest methylation level at CGI shore, CGI shelf, and opensea. In addition, the methylation values of CGI in subtype 1 samples were found to be higher than other samples (Figure 4A). This result was consistent with other studies that patients with higher CGI methylation had better prognosis.^26^, ^27^, ^28^ Then we identified the significantly different methylation CpG sites between any of the three subtypes and compared the related genes between four methylation contexts. The result showed that different methylation related genes (DMRGs) were highly specific (Figure 4E). To investigate the functional implication of the DMRGs, we performed the functional enrichment analysis of GO and KEGG for protein‐coding genes with different methylation in three subtypes. The DMRGs was enriched in cell differentiation and leukemia‐related biological processes, such as hemopoiesis and myeloid cell differentiation (Figure 5A). Despite of the difference of DMRGs between four types of genome locations, we found that DMRGs were all related to cancer pathways (Figure 5A‐D). In addition, we found that protein‐coding genes which were differently methylated between three subtypes in opensea had a contact with diabetes mellitus (Figure 5D). A previous study had found that diabetes mellitus increased the risk of developing leukemia,^29^ the result in our study also found the correlation between diabetes mellitus and leukemia.

**FIGURE 6:**
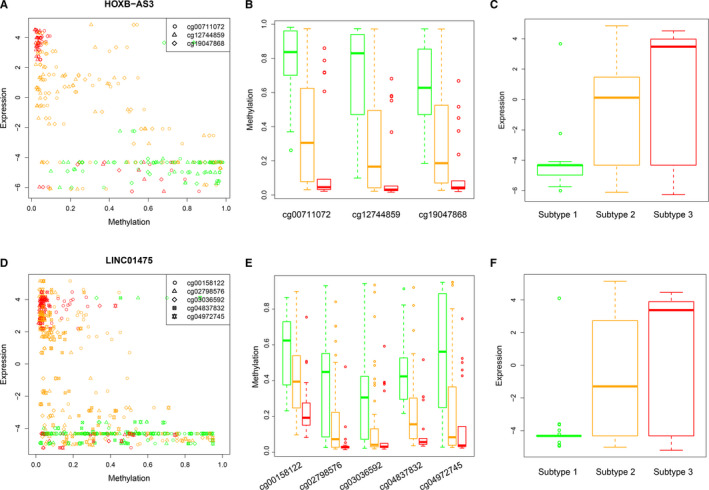
The CGI methylation and expression levels of two lncRNAs. A, The methylation of HOXB‐AS3 related CpG sites and the expression of HOXB‐AS3. Different types of points represent different CpG sites. Samples of subtypes 1, 2 and 3 are colored by green, orange and red respectively. B, The methylation of HOXB‐AS3 related CpG sites in three subtypes, including cg00711072, cg12744859, and cg19047868. Samples of subtypes 1, 2, and 3 are colored by green, orange, and red. C, The expression of HOXB‐AS3 in three subtypes. Samples of subtypes 1, 2 and 3 are colored by green, orange, and red. D‐F, LINC01475‐related methylation and expression results similar with HOXB‐AS3

Lots of studies have validated that DNA methylation in promoter CGI regulated expression of transcripts. Meanwhile, abnormal expression of mRNA/lncRNA plays roles in the occurrence and development of cancers.^30^, ^31^ Therefore, we calculated the correlation between expression of mRNA/lncRNA and methylation of each related CpG site in intervals ± 1 kb around transcription start site (TSS) of this mRNA/lncRNA, which is included in the DMRGs at CGI context. In total, we identified 29 genes whose expression whose expression was closely related to CpG sites (*R* < −0.3, *P* < .01), including 2 lncRNAs (HOXB‐AS3 and LINC01475) and 27 protein‐coding genes (Table 2). Among the 29 genes, 23, 4 and 2 genes showed highest methylation and least expression in subtype 1, 2, and 3 samples. We found that HOX family genes tend to be marker genes of subtype 1 samples with highest methylation levels, including HOXA7, HOXA9, HOXA10, HOXB3. Previous studies had revealed that high expression of HOX family genes contributed to the progression of AML.^32^, ^33^ In our study, samples of subtype 1 showed lowest expression level and best clinical prognosis. Combining these results, we supposed that aberrant promoter region hypermethylation lead to down‐regulated expression of HOX genes and further result in the progression of AML and poor prognosis. Moreover, we found that lncRNA HOXB‐AS3 showed the similar tendency of methylation and expression with HOX protein‐coding genes, which suggested that HOXB‐AS3 also may be AML marker gene (Figure 6A‐C). In addition, LINC01475 was found to be another potential AML marker lncRNA (Figure 6D‐F). Taken together, we identified a few AML marker genes with aberrant methylation pattern and regulation on transcript expression, which might be potential targets for clinical therapy.

**TABLE 2:** Protein‐coding and lncRNA genes and promoter CpG sites that regulate their expression

Gene symbol	cg
HOXB‐AS3	cg00711072, cg12744859, cg19047868
LINC01475	cg00158122, cg02798576, cg03036592, cg04837832, cg04972745
DOCK1	cg18932726
EIF5A2	cg13575298
GLB1L2	cg10700424
HAL	cg22491680
HOXA10	cg09411999
HOXA7	cg11165752, cg17642941
HOXA9	cg09411999, cg15506609, cg25999578
HOXB3	cg02311193
LDOC1	cg20104776
LRRC49	cg18517195
MAGI2	cg22280038
MARVELD2	cg05901765, cg06418871, cg06998965, cg12687157, cg17019292, cg18663063
MEIS1	cg06994420, cg09535924, cg10464312, cg12082609
MEST	cg08077673
MLXIPL	cg10092878
MPO	cg22331200
MPV17L	cg04981088
NOXA1	cg04837071
SPAG17	cg16240368
THAP10	cg18517195
TMEM204	cg10465839
TMTC1	cg10512875
UNC80	cg04100532, cg09438147, cg24938830
ZFP90	cg11625868
ZNF135	cg06454760, cg08701621, cg18430128
ZNF793	cg14732998
ZSCAN1	cg10132208, cg24368848, cg25537993

Note

Genes colored with green have highest methylation level in subtype 1 samples (lowest methylation in subtype 3, dark green; lowest methylation in subtype 2, light green). Genes colored with orange have highest methylation level in subtype 2 samples (lowest methylation in subtype 3, dark orange; lowest methylation in subtype 1, light orange). Genes colored with red have highest methylation level in subtype 3 samples (lowest methylation in subtype 2, dark red; lowest methylation in subtype 1, light red).

### 3.5 Integration of genome‐wide methylation pattern and age improved the prognostic ability for AML patients

**FIGURE 7:**
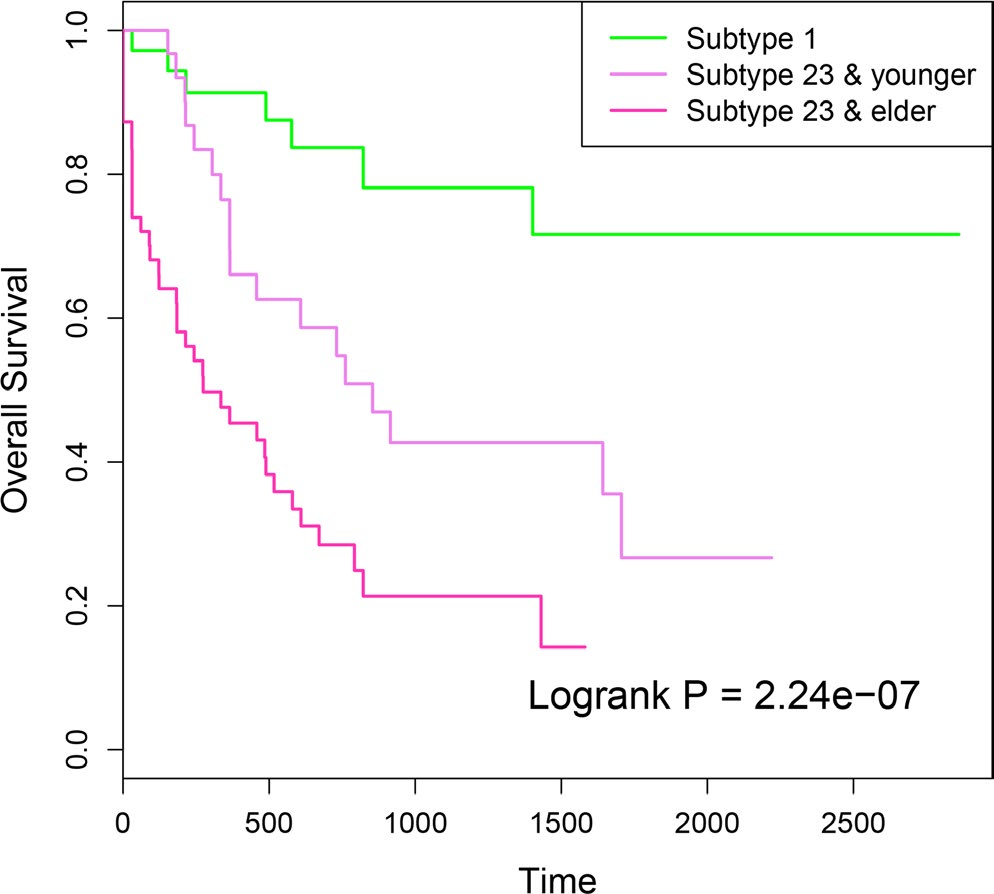
The Kaplan‐Meier survival curves for samples classified by methylation subtype and patient age

Many studies have indicated that clinical factors affect the prognosis of cancer patients.^34^ In our study, we also found that despite of the different methylation pattern, age also influenced the survival rate of AML patients. Elder patients showed significant worse prognosis than younger patients. In our previous analysis, we found that patients of subtype 1 showed significant better prognosis. In addition, patients of subtypes 2 and 3 showed significant different methylation pattern and clinical behaviors, but not significant different prognosis. Therefore, we integrated the patient age and methylation pattern, and classified samples of subtypes 2 and 3 into elder (subtype 23 & elder) and younger (subtype 23 & younger) groups. The survival analysis between subtype 1, subtype 23 & elder and subtype 23 & younger samples showed more significant survival difference (*P* = 2.24e − 07, Figure 7). Taken together, these results indicated that cancer progression was related to both molecular changes and clinical factors. Combining the molecular patterns and clinical factors will achieve more precise clinical outcomes for patients.

## 4 DISCUSSION

To investigate the role of methylation in cancers, most studies focused on the single genome context, such as CGI, while neglected CpG sites in other regions.^23^, ^24^, ^25^ In the present study, we integrated the genome‐wide CpG methylation profiles and identified the methylation subtypes for AML samples using COCA method. Analyzing the clinical outcomes for samples of the three subtypes, we found that subtype 1 showed significantly better prognosis and subtypes 2 and 3 have worse prognosis. Then we analyzed the survival rate of AML sample clusters derived from consensus clustering based on the CGI methylation profile. The result showed that although there was survival difference between the three sample clusters, the prognostic ability of CGI clusters was declined apparently. In addition, we found that methylation patterns on CGI shore, CGI shelf, and opensea contexts all showed significant difference between subtypes 1, 2, and 3. Further functional enrichments of protein‐coding genes related to four contexts were all associated with cancer progression. These results suggested that integrating the methylation profiles on genome‐wide to identify the cancer subtypes was necessary.

Through multivariate Cox regression model analysis, we found that not only methylation subtypes but also patient age had the prognostic ability. After stratifying patients into younger and elder groups, we tested the prognostic ability of methylation subtypes in the two groups with different ages. The result showed that although elder patients had worse survival rate than younger patients,^19^, ^20^, ^21^ those samples classified into subtype 1 still had better prognosis than subtypes 2 and 3. Combining patient age and methylation subtypes, we found out a better classification for AML samples. Samples of subtype 1 were with the best prognosis, followed by samples of subtypes 2 and 3 with younger age, and other samples showed the worst prognosis. Together, these results indicated that combining clinical factors and molecular variables was better prognostic strategy than single factor.

Overall, we identified the AML methylation subtypes based on the genome‐wide methylation profiles and further analyzed the different methylation patterns and clinical outcomes between these methylation subtypes. In addition, we found the different CpG sites across the three subtypes and their corresponding genes. Functional enrichment analysis revealed the cancer‐related progresses of these genes. Further expression and methylation analysis revealed the marker genes of AML subtypes. These findings provide additional useful data for the development of clinical therapeutic targets against AML.

## CONFLICT OF INTEREST

The authors declare no competing interests.

## AUTHORS' CONTRIBUTIONS

XL and WW conceived and designed the experiments. HG, XH, QL, XL, YW, YT, XC, and YG analyzed the data. XL and WW wrote the manuscript. All authors have read and approved the final manuscript.

## ETHICS APPROVAL AND CONSENT TO PARTICIPATE

Not applicable. All analyses were based on previously published studies, thus no ethical approval and patient consent are required.

## Data Availability

The datasets used and analyzed during the current study are available from the corresponding author on reasonable request.

## References

[1] DiNardo CD , Stone RM , Medeiros BC . Novel therapeutics in acute myeloid leukemia. Am Soc Clin Oncol Educational Book. 2017;37:495‐503. 28561688 10.1200/EDBK_175401

[2] Döhner H , Estey EH , Amadori S , et al. Diagnosis and management of acute myeloid leukemia in adults: recommendations from an international expert panel, on behalf of the European LeukemiaNet. Blood. 2010;115(3):453‐474. 19880497 10.1182/blood-2009-07-235358

[3] Patel JP , Gönen M , Figueroa ME , et al. Prognostic relevance of integrated genetic profiling in acute myeloid leukemia. N Engl J Med. 2012;366(12):1079‐1089. 22417203 10.1056/NEJMoa1112304 PMC3545649

[4] Vardiman JW , Thiele J , Arber DA , et al. The 2008 revision of the World Health Organization (WHO) classification of myeloid neoplasms and acute leukemia: rationale and important changes. Blood. 2009;114(5):937‐951. 19357394 10.1182/blood-2009-03-209262

[5] Jones PA . Functions of DNA methylation: islands, start sites, gene bodies and beyond. Nat Rev Genet. 2012;13(7):484‐492. 22641018 10.1038/nrg3230

[6] Jung N , Dai B , Gentles AJ , Majeti R , Feinberg AP . An LSC epigenetic signature is largely mutation independent and implicates the HOXA cluster in AML pathogenesis. Nat Commun. 2015;6:8489. 26444494 10.1038/ncomms9489 PMC4633733

[7] Moore LD , Le T , Fan G . DNA methylation and its basic function. Neuropsychopharmacology. 2013;38(1):23‐38. 22781841 10.1038/npp.2012.112 PMC3521964

[8] Bozic T , Frobel J , Raic A , et al. Variants of DNMT3A cause transcript‐specific DNA methylation patterns and affect hematopoiesis. Life Sci Alliance. 2018;1(6):e201800153. 30582132 10.26508/lsa.201800153 PMC6293073

[9] Skvortsova K , Stirzaker C , Taberlay P . The DNA methylation landscape in cancer. Essays Biochem. 2019;63(6):797‐811. 31845735 10.1042/EBC20190037 PMC6923322

[10] Visone R , Bacalini MG , Franco SD , et al. DNA methylation of shelf, shore and open sea CpG positions distinguish high microsatellite instability from low or stable microsatellite status colon cancer stem cells. Epigenomics. 2019;11(6):587‐604. 31066579 10.2217/epi-2018-0153

[11] Jeltsch A , Jurkowska RZ . New concepts in DNA methylation. Trends Biochem Sci. 2014;39(7):310‐318. 24947342 10.1016/j.tibs.2014.05.002

[12] Lea AJ , Vockley CM , Johnston RA , et al. Genome‐wide quantification of the effects of DNA methylation on human gene regulation. eLife. 2018;7(7):1‐27. 10.7554/eLife.37513 PMC6303109 30575519

[13] Jones PA , Baylin SB . The fundamental role of epigenetic events in cancer. Nat Rev Genet. 2002;3(6):415‐428. 12042769 10.1038/nrg816

[14] Galm O , Herman JG , Baylin SB . The fundamental role of epigenetics in hematopoietic malignancies. Blood Rev. 2006;20(1):1‐13. 16426940 10.1016/j.blre.2005.01.006

[15] Hoadley KA , Yau C , Wolf DM , et al. Multiplatform analysis of 12 cancer types reveals molecular classification within and across tissues of origin. Cell. 2014;158(4):929‐944. 25109877 10.1016/j.cell.2014.06.049 PMC4152462

[16] Wilkerson MD , Hayes DN . ConsensusClusterPlus: a class discovery tool with confidence assessments and item tracking. Bioinformatics. 2010;26(12):1572‐1573. 20427518 10.1093/bioinformatics/btq170 PMC2881355

[17] da Huang W , Sherman BT , Lempicki RA . Bioinformatics enrichment tools: paths toward the comprehensive functional analysis of large gene lists. Nucleic Acids Res. 2009;37(1):1‐13. 19033363 10.1093/nar/gkn923 PMC2615629

[18] Dalai W , Matsuo E , Takeyama N , Kawano J , Saeki K . CpG site DNA methylation patterns reveal a novel regulatory element in the mouse prion protein gene. J Veterinary Med Sci. 2017;79(1):100‐107. 10.1292/jvms.16-0390 PMC5289245 27666463

[19] Mamtani R , Wang XV , Gyawali B , et al. Association between age and sex and mortality after adjuvant therapy for renal cancer. Cancer. 2019;125(10):1637‐1644. 30620389 10.1002/cncr.31955 PMC6486432

[20] Roviello G , Corona SP , Aieta M , Roudi R . Influence of age and the gleason score in the choice of novel hormonal therapies before and after chemotherapy. Cancer Biother Radiopharm. 2019;34(3):141‐146. 30620216 10.1089/cbr.2018.2702

[21] Santoro N , Labopin M , Ciceri F , et al. Impact of conditioning intensity on outcomes of haploidentical stem cell transplantation for patients with acute myeloid leukemia over 45 years of age. Cancer. 2019 125(9):1499‐1506. 30620383 10.1002/cncr.31941

[22] Negrini S , Gorgoulis VG , Halazonetis TD . Genomic instability–an evolving hallmark of cancer. Nat Rev Mol Cell Biol. 2010;11(3):220‐228. 20177397 10.1038/nrm2858

[23] Qiao B , Zhang Z , Li Y . Association of MGMT promoter methylation with tumorigenesis features in patients with ovarian cancer: a systematic meta‐analysis. Molecular Genetics Genomic Med. 2018;6(1):69‐76. 10.1002/mgg3.349 PMC5823672 29195029

[24] Lounglaithong K , Bychkov A , Sampatanukul P . Aberrant promoter methylation of the PAQR3 gene is associated with prostate cancer. Pathol Res Pract. 2018;214(1):126‐129. 29122400 10.1016/j.prp.2017.10.010

[25] Ma Y , Chen Y , Petersen I . Expression and promoter DNA methylation of MLH1 in colorectal cancer and lung cancer. Pathol Res Pract. 2017;213(4):333‐338. 28214209 10.1016/j.prp.2017.01.014

[26] Kelly AD , Kroeger H , Yamazaki J , et al. A CpG island methylator phenotype in acute myeloid leukemia independent of IDH mutations and associated with a favorable outcome. Leukemia. 2017;31(10):2011‐2019. 28074068 10.1038/leu.2017.12 PMC5537054

[27] Yanokura M , Banno K , Adachi M , Aoki D , Abe K . Genome‐wide DNA methylation sequencing reveals miR‐663a is a novel epimutation candidate in CIMP‐high endometrial cancer. Int J Oncol. 2017;50(6):1934‐1946. 28440489 10.3892/ijo.2017.3966 PMC5435325

[28] Bae JM , Kim JH , Kwak Y , et al. Distinct clinical outcomes of two CIMP‐positive colorectal cancer subtypes based on a revised CIMP classification system. Br J Cancer. 2017;116(8):1012‐1020. 28278514 10.1038/bjc.2017.52 PMC5396110

[29] Castillo JJ , Mull N , Reagan JL , Nemr S , Mitri J . Increased incidence of non‐Hodgkin lymphoma, leukemia, and myeloma in patients with diabetes mellitus type 2: a meta‐analysis of observational studies. Blood. 2012;119(21):4845‐4850. 22496152 10.1182/blood-2011-06-362830 PMC3367891

[30] Gupta A , Ahmad MK , Mahndi AA , Singh R , Pradeep Y . Promoter methylation and relative mRNA expression of the p16 gene in cervical cancer in North Indians. Asian Pac J Cancer Prev. 2016;17(8):4149‐4154. 27644676

[31] Wang FL , Yang Y , Liu ZY , Qin Y , Jin T . Correlation between methylation of the p16 promoter and cervical cancer incidence. Eur Rev Med Pharmacol Sci. 2017;21(10):2351‐2356. 28617556

[32] Alharbi RA , Pettengell R , Pandha HS , Morgan R . The role of HOX genes in normal hematopoiesis and acute leukemia. Leukemia. 2013;27(5):1000‐1008. 23212154 10.1038/leu.2012.356

[33] Rejlova K , Musilova A , Kramarzova KS , et al. Low HOX gene expression in PML‐RARalpha‐positive leukemia results from suppressed histone demethylation. Epigenetics. 2018;13(1):73‐84. 29224413 10.1080/15592294.2017.1413517 PMC5836981

[34] Zhou M , Wang X , Shi H , et al. Characterization of long non‐coding RNA‐associated ceRNA network to reveal potential prognostic lncRNA biomarkers in human ovarian cancer. Oncotarget. 2016;7(11):12598‐12611. 26863568 10.18632/oncotarget.7181 PMC4914307

